# Cyanobacteria and Algae Blooms: Review of Health and Environmental Data from the Harmful Algal Bloom-Related Illness Surveillance System (HABISS) 2007–2011

**DOI:** 10.3390/toxins7041048

**Published:** 2015-03-27

**Authors:** Lorraine C. Backer, Deana Manassaram-Baptiste, Rebecca LePrell, Birgit Bolton

**Affiliations:** 1 National Center for Environmental Health, Centers for Disease Control and Prevention, 4770 Buford Highway NE, Chamblee, GA 30341, USA; 2 American Cancer Society, 250 Williams Street, Quad 6C, Atlanta,‎ GA 30303, USA; E-Mail: deana.baptiste@cancer.org; 3 Virginia Department of Health, 109 Governor Street, Richmond, VA 23219, USA; E-Mail: rebecca.leprell@vdh.virginia.gov; 4 International Trachoma Initiative, the Task Force for Global Health, 325 Swanton Way, Decatur, GA 30030, USA; E-Mail: bbolton@taskforce.org

**Keywords:** cyanobacteria, harmful algae, blooms, disease surveillance

## Abstract

Algae and cyanobacteria are present in all aquatic environments. We do not have a good sense of the extent of human and animal exposures to cyanobacteria or their toxins, nor do we understand the public health impacts from acute exposures associated with recreational activities or chronic exposures associated with drinking water. We describe the Harmful Algal Bloom-related Illness Surveillance System (HABISS) and summarize the collected reports describing bloom events and associated adverse human and animal health events. For the period of 2007–2011, Departments of Health and/or Environment from 11 states funded by the National Center for Environmental Health (NCEH), Centers for Disease Control and Prevention contributed reports for 4534 events. For 2007, states contributed 173 reports from historical data. The states participating in the HABISS program built response capacity through targeted public outreach and prevention activities, including supporting routine cyanobacteria monitoring for public recreation waters. During 2007–2010, states used monitoring data to support196 public health advisories or beach closures. The information recorded in HABISS and the application of these data to develop a wide range of public health prevention and response activities indicate that cyanobacteria and algae blooms are an environmental public health issue that needs continuing attention.

## 1. Introduction

Algae and cyanobacteria are present in all aquatic environments, and these organisms produce some of the most potent natural toxins known. We do not have a good sense of the extent of human and animal exposures to these organisms or their toxins, nor do we understand the public health impacts from acute exposures associated with recreational activities or chronic exposures associated with drinking water. To support public health decision-making about health risks from exposure to cyanobacteria and algae blooms and associated toxins, various efforts have been undertaken to collect and assess data describing the blooms, exposures, and associated human and animal health outcomes.

Efforts to understand algae and cyanobacteria blooms and the full spectrum of public health effects were initially supported by the Centers for Disease Control and Prevention’s Cooperative Agreements (Program Announcement Number 98019 (1998); Program Announcement Number 03102 (2003); CDC-RFA-EH08-801 (2009)). This funding was intended to support state responses to the purported adverse human health and ecologic effects associated with the presence of the dinoflagellate, *Pfistieria psicicida* in the Chesapeake Bay [1]. Specifically, original goals for the NCEH funding were to develop a case definition for disease associated with exposure to *P. piscicida* and any toxins it produced, conduct health surveillance, conduct analytic studies, and develop a biomarker of exposure/effect.

Eventually, it was determined that *P. piscicida* did not pose a substantive, definable human health threat, e.g., [2]. However, states identified potential public health issues associated with exposures to toxins produced by other types of algae (e.g., brevetoxins produced by the marine dinoflagellate *Karenia brevis*, the organism responsible for Florida red tides, and microcystins produced by several species of cyanobacteria, the organisms responsible for many green scums on fresh waters). In response, state activities supported by CDC funding expanded to include human and animal morbidity and mortality associated with exposure to any cyanobacteria or harmful algae bloom.

During this time, the National Center for Environmental Health (NCEH), CDC created, in collaboration with state partners, the Harmful Algal Bloom-related Illness Surveillance System (HABISS). The goals for HABISS were: (1) Describe the temporal and geographic distribution of cyanobacteria and algae blooms and (2) Describe the suspected human and animal morbidity and mortality associated with bloom events. HABISS was uniquely designed to capture, in one database, information describing suspected adverse human and animal health effects and environmental information about blooms as potential sources of exposure. A review of the HABISS data collection system was published by Glynn and Backer [3].

Various other efforts captured the public health consequences associated with cyanobacteria and algae blooms. Reports of health events associated with exposure to cyanobacteria, algae, and related toxins have been captured in CDC’s National Outbreak Reporting System (NORS) [4]. Hilborn *et al.* [5] compiled a summary of 11 outbreaks involving 58 persons reported to NORS between 2009 and 2010.

Animal morbidity and mortality is an indicator for human health. To summarize what is currently known about cyanobacteria poisonings of pet dogs, Backer *et al.* [6] reviewed information collected from the following sources: HABISS; retrospective case files from a large, regional veterinary hospital in California; and publicly available information including scientific and medical manuscripts; written media; and web-based reports from pet owners, veterinarians, and other individuals. The authors summarized 231 discreet events and identified 368 cases of cyanotoxin poisonings of pet dogs throughout the U.S. between the late 1920s and 2012.

In the present paper, we describe HABISS and summarize the collected reports describing bloom events and suspected adverse health effects associated with exposure to freshwater cyanobacteria blooms or marine HABs and their associated toxins. We also discuss other public health promotion activities conducted by states that received funding from NCEH to address cyanobacteria and algae blooms.

## 2. Materials and Methods

### 2.1. HABISS

#### 2.1.1. Partners

NCEH partnered with state representatives from the 11 funded states (FL, IA, MD, MA, NC, NY, OR, SC, VA, WA, WI; note that not all states were funded for the entire period from 1998 to 2013) and representatives from the University of Miami and Wright State University to develop the initial data collection instruments for HABISS. Data collection ended in 2013 when funding ended.

#### 2.1.2. Electronic Platform

HABISS was an active surveillance system that operated on NCEH’s secure platform, the Rapid Data Collector (RDC) [3]. The RDC tool was designed by NCEH specifically for rapid survey design and data collection; and HABISS was the first RDC tool supported for long-term surveillance. Protected by a secure data network (SDN), state users could choose to enter, edit, and save data for subsequent sessions.

#### 2.1.3. HABISS Surveillance

HABISS was a passive surveillance activity for tracking reports of human and animal morbidity and mortality from exposures to cyanobacteria and algae blooms. Reports often triggered active surveillance activities, including direct follow-up on events or reports of morbidity/mortality in the media.

#### 2.1.4. Data Elements

HABISS allowed contributors to input several key indicators (e.g., dates, agency contact information, state codes, route of exposure, patient’s chief complaint). HABISS prompted users to report data elements to describe suspected or confirmed human or animal illnesses. The data elements were parallel for reporting adverse health effects for humans and animals and included contact location with the HAB or cyanobacteria bloom, identifying information for the potential case (which was not shared with CDC), demographics of the potential case, environmental information describing the water body, exposure information, signs and symptoms, medical review, laboratory analyses, and case assessment and follow-up. We worked with our state partners to ensure they did not enter duplicate cases into the system.

HABISS contributors were requested to report key data elements for a bloom report, which included water sample collection methods, analytic methods and test results, taxonomy, toxin identification and concentration, and geographic coordinates via a live link to Google Maps^©^. Human and animal morbidity and mortality reports were linked to each other and linked to data collected on relevant blooms.

There were few established case definitions to use for this surveillance effort see, for example, [7,8]. State contributing data used categories of confidence that the case or outbreak was bloom-related defined as follows: (1) A *suspect* case had exposure to water or seafood with a confirmed bloom AND onset of associated signs and symptoms within a reasonable time after exposure AND without identification of another cause of illness; (2) A *probable* case met criteria for suspect case AND there is laboratory documentation of bloom toxins in the water; and (3) A *confirmed* case met criteria for probable case combined with a professional judgment based on medical review. Also, a case may be defined as confirmed by meeting criteria for a probable case AND having documentation of a bloom-related toxin in a clinical specimen.

#### 2.1.5. Contributors

The 11 funded states (FL, IA, MD, MA, NC, NY, OR, SC, VA, WA, and WI) and four additional states (CA, KS, MN, and MT) contributed data to HABISS.

#### 2.1.6. Data Sources

The states used various methods to gather data describing cyanobacteria and harmful algae blooms and associated health effects. Many states previously identified cyanobacteria and harmful algae blooms as an ongoing public health issue and had established continual or seasonal monitoring programs. Environmental data, which may include water samples, captured in HABISS were categorized as follows: (1) Routine monitoring at public water bodies used for recreational purposes or water bodies with a history of blooms to monitor conditions that could be used to predict blooms; (2) Health event response, *i.e.*, collection of data in response to a human or animal illness or death following exposure to that water body, even in absence of a visible bloom; (3) Bloom report response, *i.e.*, collection of data from a water body reported to be blooming. The bloom could be reported by residents after noticing an unusual appearance or odor emanating from a community water body, local health officials, environmental regulatory agency staff, local or state park staff, watershed organizations, or other community groups; or (4) Fish kill response, *i.e.*, collection of data in response to a report of sick or dead fishes in or around the shoreline of a water body.

All contributing states conducted investigations if there was a case or outbreak of illness reported after exposure to recreational waters. These follow-up investigations included gathering environmental data (sometimes from established monitoring programs), water sample collection and analysis, and interviewing potential cases, when possible, by telephone or in person to ascertain exposure and health effects information. Owners whose pet dog or other domestic animal became ill or died after exposure to water with a suspected bloom or veterinarians who treated these animals provided health-related data during telephone interviews with investigators.

Several other sources of information for bloom-related health effects contributed to HABISS. NCEH monitored the National Poison Data System for reports of specific diseases, such as ciguatera fish poisoning and shared these alerts with appropriate states for follow-up. Other sources of data included the national and state notifiable disease reporting systems [9]. NCEH monitored media reports using Google Alerts^©^, and states conducted relevant follow-up activities when bloom-related cases of illness were identified.

#### 2.1.7. Data Access and Download

Data contributors had access to their state’s data as well as to data from other states with which they had established data sharing agreements. Data could be exported into Access^©^, Excel^©^, or XML^©^ for analysis.

## 3. Results

For the period of 2007–2011, Departments of Health and/or Environment from 11 states funded by NCEH (Florida, Iowa, Maryland, Minnesota, New York, North Carolina, Oregon, Virginia, Washington, and Wisconsin) contributed reports for 4534 events. For 2007, states contributed 173 reports from historical data. The states also reported 458 cases of suspected and confirmed human bloom-associated illnesses and 175 animal morbidity and mortality events.

Although most of the contributing states conducting routine monitoring of public recreation waters for blooms, other states collected and documented bloom events only if there were reports of visible blooms, blooms with harmful algae or cyanobacteria identified, fish mortalities, or illnesses in people or animals following exposure to the water body.

The majority of reports (4245, 94%) represented routine monitoring. A summary of the number of reports and the reasons for the associated data collection by year is in Table 1.

**Table 1:** The number of reports recorded in Harmful Algal Bloom-related Illness Surveillance System (HABISS) from 2007 to 2011, by year, and the reason why the data were collected.

Year	Reason for Bloom-Related Data Collection (Percent by Year)				
	Routine Monitoring	Bloom Report Response	Health Event Response	Fishkill Response	Total Reports
2007	167 (96)	1 (<1)	5 (3)	0	173
2008	509 (90)	7 (1)	41 (7)	8 (1)	565
2009	1344 (93)	55 (4)	28 (19)	23 (2)	1450
2010	977 (94)	25 (2)	19 (2)	16 (2)	1037
2011	1248 (95)	31 (2)	20 (52)	10 (1)	1309
**Total Reports**	4245	119	113	57	4534

### 3.1. Environmental Reports

Freshwater sources, which included lakes, rivers, streams, and ponds, were the most frequently reported type of water body (*n* = 3499, or 77% of the reports). Of the remaining reports, brackish (*i.e*., a mixture of marine and fresh water) water bodies were identified in 973 (21%); marine waters in 82 (2%) and unknown water bodies in 172 (4%) of the reports.

### 3.2. Cyanobacteria, Algae, and Toxin Testing

Contributors identified cyanobacteria, the most common type of organism reported, in 1690 of 2323 (73%) samples analyzed for organism taxonomy*.* States most commonly reported *Anabaena* spp. (454, 20% of samples) followed by *Aphanizomenon* spp. (164, 7% of samples), and *Microcystis* spp. (165 or 7% of samples). Cyanobacteria cell counts ranged from 12,060 cells/mL of water (*Aphanizomenon* spp.) in a sample collected in response to a health event to 40,106,667 cells per mL of water (*Anabaena flos-aquae*) in a sample collected for routine monitoring (Table 2).

**Table 2:** Maximum and mean cell counts for cyanobacteria species listed by reason for sample collection. Samples collected in response to fish kills were not analyzed for *Microcystis* spp.

Species	Reason for Sample Collection (Cells/mL)			
	Monitoring	Health Event Response	Bloom Response	Fish Kill
*Anabaena* spp.	Max: 40,107,000	Max: 731,000	Max: 4,231,000	Max: 472,000
	Mean: 516,000	Mean: 96,000	Mean: 294,000	Mean: 267,000
	*N* = 360	*N* = 12	*N* = 22	*N* = 4
*Aphanizomenon* spp.	Max: 16,912,000	Max: 12,000	Max: 172,000	Max: 19,146,000
	Mean: 533,000	Mean: 6300	Mean: 85,500	Mean: 4,939,000
	*N* = 102	*N* = 6	*N* = 14	*N* = 4
*Microcystis* spp.	Max: 6,742,000	Max: 614,000	Max: 230,000	Max: NA
	Mean: 212,000	Mean: 194,000	Mean: 60,000	Mean: NA
	*N* = 117	*N* = 14	*N* = 9	*N* = 4

Other identified species included diatoms, dinoflagellates, prorocetrales, and raphidophyceans. Thirty-nine (2%) samples contained multiple species, and 367 (16%) of samples were not analyzed for cyanobacteria or algae species.

States reported toxin testing in 3301 reports (Table 3). Microcystins were the most common toxin and were identified in 2629 (80%) samples followed by saxitoxins (311 samples, 9%) anatoxin-a (246 samples, 7%). Domoic acid was the most commonly reported marine toxin (31 samples, 1%). The toxins maitotoxin and prymnesin were not found in any samples.

HABISS contributors recorded data related to cyanobacteria blooms in fresh waters for 439 water samples collected in response to a report of an ongoing bloom and 120 water samples collected in response to an adverse health event involving people or animals. Contributors also recorded data for 97 water samples collected in response to fish kills that occurred in fresh or brackish waters. A summary of cyanobacteria cell counts and toxin concentrations for samples collected in response to these events is in Table 4.

**Table 3:** Toxins identified in the first water sample collected (2007–2011) by type of water sample.

Toxin	Water Type				
	Fresh	Brackish	Marine	Unknown	Total (%)
Anatoxin	243	2	0	1	246 (7)
Azaspiracid	0	0	1	0	1 (<1)
Brevetoxoins	0	3	0	0	3 (<1)
Cylindrospermopsin	4	0	0	0	4 (<1)
Domoic Acid	0	0	31	0	31 (1)
Karlotoxins	0	3	1	0	4 (<1)
Microcytins Total	2629	35	2	10	2676 (81)
Microcytsin LR	21	0	0	0	21 (1)
Okadaic Acid	1	2	0	0	3 (<1)
Saxitoxins	296	1	11	3	311 (9)
Unidentified Toxin	0	1	0	0	1 (<1)
**Total**	3194	47	46	14	3301

**Table 4:** Cyanobacteria cell counts and toxin concentrations in water samples collected in response to a health event, fish kill, or report of an ongoing bloom. Values are median (range).

Water Sample Parameter	Reason for Water Sample Collection		
	Response to a Human or Animal Health Event	Response to a Fish Kill	Response to a Report of a Bloom
*Cyanobacteria Cell Counts (cells/mL)*			
*Anabaena* spp.	12,700 (251–7,600,000) (*N* = 13)	34,000 (184–190,000) (*N* = 14)	19,000 (87–4,231,033) (*N* = 79)
*Aphanizomenon* spp.	NR ^1^	41,000 (9000–9,500,000) (*N* = 7)	26,000 (230–172,000) (*N* = 61)
*Cylindrospermopsis* spp.	1700 (*N* = 1)	NR	14,017 (34–28,000) (*N* = 2)
*Lyngbya* spp.	206 (*N* = 1)	98,400 (*N* = 1)	NR
*Microcystis* spp.	27,000 (81–6,100,000) (*N* = 16)	8000 (1200–614,000) (*N* = 4)	14,300 (60–253,849) (*N* = 36)
*Oscillatoria* spp.	NR	32,000 (6500–521,000) (*N* = 5)	NR
*Planktothrix* spp.	140 (26–245) (*N* = 2)	NR	NR
*Pseudoanabaena* spp.	119 (28–210) (*N* = 2)	930 (105–116,000) (*N* = 3)	NR
*Woronichia* spp.	NR	NR	13,000 (11,000–23,000) (*N* = 3)
*Toxin Concentrations (µg/L)*			
Anatoxin	0.75 (0–500) (*N* = 12)	NR	0.05 (0–3,302,000) (*N* = 15)
Brevetoxins	NR	0.0 (0.0–1.0) (*N* = 7) ^2^	NR
Cylindrospermopsin	0.5 (0–0.5) (*N* = 6)	NR	0.1 (0–9.0) (*N* = 5)
Microcystins total	15 (0–700) (*N* = 19)	0.06 (0.0–307) (*N* = 22)	0.9 (0–1385) (*N* = 140)
Microcystin-LR	1.3 (1–249) (*N* = 9)	NR	0.8 (0.2–1,120,000) (*N* = 16)

^1^: Not reported; ^2^: *Chatonella* spp. identified but not quantified.

### 3.3. Animal Health Events

Animal illness reports included individual case reports for domestic animals and wildlife and case reports involving flocks of birds or schools of fish. During 2007–2011, states reported 175 events of animal morbidity or mortality. Of these, 93 (53%) were described as probable or suspected, and 7 (4%) were described as confirmed bloom-related events. There were 95 reports of fish mortalities; however, fewer than half (*n* = 35, 37%) were reported as bloom-related. Low dissolved oxygen concentration was the cause of fish mortalities in 30 (32%) events. Among the 11 reported cases of livestock mortalities, nine were cows that died in a single event in Montana after drinking water from a lake with a visible bloom. However, no water testing confirmed the presence of harmful algae, cyanobacteria, or toxins. There was one case report describing bird mortalities, but no environmental data were collected during the event.

Among domestic animal reports, canine poisonings were the most frequent (*n* = 67, 38%). Thirty-eight (57%) of the canine poisonings were fatal. Common reported routes of exposure for dogs included dermal contact (*n* = 36, 54%) and inhalation/ingestion from swimming in water with an ongoing bloom (*n* = 15, 22%). Gastrointestinal symptoms (e.g., vomiting, foaming at the mouth) were most frequently reported symptoms in dogs (*n* = 29, 43%), followed by lethargy (*n* = 12, 18%) and neurologic symptoms such as stumbling and behavior changes (*n* = 6, 9%).

The majority of canine-related reports (58% of 67% or 87%), the 11 cattle mortality reports, and the single bird mortality report were associated with exposures to fresh waters. Most of the fish-kill-related reports (61% of 95%, or 64%) were associated with brackish waters.

Water samples were collected in response to 74 (42%) animal morbidity or mortality reports; however, 37 (50%) of these events were reported as cyanobacteria- or algae-related even though no cells or toxins were identified. The presence of cyanobacteria in water samples was reported for 24 (32%) events. Of the events accompanied by data on water samples, anatoxin-a was identified in 14 (19%) samples and microcystins were identified in 13 (18%) samples. Of the 67 canine mortalities, anatoxin exposure was implicated in 12 (18%), microcystin poisoning was identified in 3 (4%), and no specific toxin was noted for 5 (7%) cases. For the canine poisonings, toxin levels were measured in water samples collected in response to the event.

### 3.4. Human Health Events

During the period 2007–2011, states contributed 584 case reports of human illnesses associated with exposures to cyanobacteria or algae. Of these, 253 (40%) were described as probable or suspect and 219 (38%) met the criteria for confirmed cases. Of the 456 case reports with relevant data (two reports did not provide source of exposure), food was identified as the source of exposure in 273 (60%) reports and water was identified as the source of exposure in 183 (40%) reports. Of the reports identifying water-related exposures, freshwaters 176 (96%) were the most common, followed by marine waters (4, 2%) and brackish water (1, <1%).

Of the reports identifying food as the source of exposure, 248 (91%) reported ciguatera fish poisoning or poisoning by other toxins in seafood, including saxitoxin (13, 5%) and brevetoxin (2, <1%). The first or primary symptom was noted for only 74 (27%) food-related human cases and included gastrointestinal symptoms for 35 (47%) cases and neurologic symptoms for 22 (28%) cases. For the 207 reports in which the food was specified, the source of exposure was finfish for 185 (89%) cases and shellfish for 22 (11%) cases.

The majority of ciguatera fish poisoning cases (211, 85%) met the confirmed case definition, and the remaining 37 (15%) cases were reported as suspect or probable. Confirmed cases that occurred in states where physicians are required to report ciguatera fish poisoning (e.g., Florida and Washington) met the reporting state’s case definitions. Confirmed cases that occurred in other states met the HABISS case definition: symptoms compatible the ciguatera fish poisoning (e.g., neurologic symptoms, including paresthesias of the extremities or metallic taste; gastrointestinal distress; hypotension with bradycardia), acute onset of illness within hours after eating a suspect fish (e.g., grouper, barracuda), and either verification of the toxin in a leftover meal remnant or professional medical judgment following medical review. A recent review provides an update of ciguatera signs and symptoms, treatments, and long-term sequelae [10].

Contributors reported 181 probable and confirmed cases of illness related to sources other than food. The reported health effects included 89 (49%) reports of rash from exposure to unknown toxin, 28 (16%) reports of microcystin poisoning, and other reports (27, 14%) of gastrointestinal or respiratory illness for which there were no confirmed toxin exposures (see Table 5). The primary or first symptom reported by probable and confirmed non-food-related cases (181 reports) included dermatologic effects such as rash, itching, and blisters (*n* = 93, 51%); gastrointestinal symptoms such as nausea and vomiting (*n* = 25, 19%); neurologic effects such as weakness and confusion (*n* = 11, 6%); and other general symptoms such as fatigue and fever (*n* = 10, 6%). For the subgroup of cases (*n* = 174) where non-food exposure was reported, most (*n* = 157, 90%) exposures occurred during recreational activities such as swimming, using personal water craft, or boating; and 17 (10%) cases occurred during occupational activities, such as water sample collection. Dermal contact (*n* = 119, 66%) and inhalation (*n* = 51, 28%) were the most common routes of non-food related exposures for these cases.

**Table 5:** Suspected and confirmed cases of human illnesses following exposure to cyanobacteria or algae (2007–2011).

Human Illness	Number of Cases (%)
Ciguatera fish poisoning	248 (54)
Rash from unknown organism or toxin	89 (19)
Illness from unknown organism or toxin	49 (11)
Microcystin poisoning	28 (6)
Other cyanobacteria- or algae-related illness not specified in HABISS	27 (6)
Paralytic shellfish poisoning (saxitoxins)	13 (3)
Neurotoxic shellfish poisoning (brevetoxins)	2 (<1)
Anatoxin poisoning	1 (<1)
Amnesic shellfish poisoning (domoic acid)	1 (<1)
Total	458

Of the 181 reports of non-food related human illness reports, 80 (44%) were linked to environmental data, for which 55 (40%) included water sample analysis for cyanobacteria, algae and/or toxins. Of the 52 case reports associated with exposure to cyanobacteria, 28 (54%) water samples contained *Microcystis spp*., 20 (38%) contained *Anabaena spp*., and 4 (8%) contained *Aphanizomenon spp*. Toxin test results were included in 27 of these reports; anatoxin, microcystins, and cylindrospermopsins were found in 22 (81%), 4 (15%), and 1 (4%) samples, respectively.

### 3.5. Public Health Actions Supported by the HABISS Program

One of the goals of HABISS was to accumulate data to support public health activities to reduce future morbidity and mortality resulting from exposure to harmful algal blooms. States participating in the HABISS program built response capacity through targeted public outreach and prevention activities. Some states also used resources provided by NCEH to increase or implement routine cyanobacteria monitoring for public recreation waters to prevent human and animal exposures to potentially hazardous blooms. During 2007–2010, states used these monitoring data to support decisions to issue 196 public health advisories or beach closures.

A sick or dead animal may be the first indication that a water body has an ongoing cyanobacteria or algae bloom. NCEH used information from HABISS to create a tool kit of materials (e.g., veterinary reference card, animal health alert poster) to raise awareness among veterinarians and the public about risks to pets from cyanobacteria. Between 2009 and 2010, NCEH distributed 2000 fact cards and 1500 posters to state partners. The tool kit also includes a physician reference card. Materials will be available as part of the updated CDC Drinking Water Advisory Communication Toolbox [11]. 

Examples of state activities, partnerships, and protocols developed using resources provided by HABISS is in Table 6.

**Table 6:** State activities, partnerships, and protocols that were developed using resources provided by HABISS. URL links to websites and materials are provided when available.

State Entity that Received Funding from HABISS	Activities, Partnerships, and Protocols Supported by HABISS
Florida Department of Health (FDOH)	•Supplemented ongoing public health surveillance (*i.e*., reportable conditions using a system called MERLIN, foodborne disease surveillance, and events identified through the Poison Information Center) via the Aquatic Toxins Disease Prevention Program (ATDPP). Case reports and complaints associated with exposure to cyanobacteria blooms and reports of respiratory illnesses associated with exposures to airborne brevetoxins during Florida red tide events were recorded in HABISS. •Used the Electronic Surveillance System for Early Notification of Community Epidemics (ESSENCE) to provide early warning of cyanobacteria- and algae-related illness outbreaks, rapidly detect and report cyanobacteria and algae blooms, and identify bloom events with potential public health significance. •Made information available on website: http://www.floridahealth.gov/environmental-health/aquatic-toxins/index.html.
Iowa: Harmful Algal Bloom Program, Iowa Department of Public Health (IHAB)	•Implemented a cyanobacteria bloom advisory for Iowa during the 2011 monitoring season, and the IDPH temporarily designated suspected or confirmed exposures to microcystins as a reportable condition. The designation will continue to be used during future bloom seasons. •In 2010, collaborated with IDNR to launch an improved version of their interactive mapping application to allow visitors to the site to view current swimming advisories and water quality information, including the most recent sample analyzed for microcystins, for state park beaches. The website allows the public to contact IDPH with questions about beach water quality and to report a bloom or suspected bloom exposure.
Massachusetts Bureau of Health, Massachusetts Department of Public Health BEH/MDPH	•Used active surveillance to improve bloom-related symptom and illness reporting. •Provided bloom data that served as guidance for local health officials, other government officials, and community members to address health concerns related to blooms. For example, during the 2009 and 2010 bloom seasons, BEH used HABISS data to support issuing 23 and 24 health advisories, respectively, for public recreational waters affected by cyanobacteria blooms.
Maryland Department of Health and Mental Hygiene MDHMH)	•Expanded a web-based bloom outreach program to health care professionals through a series of Grand Rounds. •Linked monitoring data with the MD Healthy Beaches program as part of risk communication and public outreach efforts.
New York State Department of Health	•Conducted outreach activities to expand knowledge and awareness of harmful cyanobacteria and algae blooms. •http://www.dec.ny.gov/outdoor/64824.html •http://www.nyhealth.gov/environmental/water/drinking/bluegreenalgae.htm •Provided park rangers, lifeguards, and victims of cyanobacteria exposures information, fact sheets, water sampling and analysis, and guidance for bloom response. •Collaborated with the Citizen Statewide Lake Assessment Program, a citizen-based monitoring program run by the NYS Department of Environmental Conservation and the NYS Federation of Lake Associations.
North Carolina	•Provided information on website: http://epi.publichealth.nc.gov/oee/a_z/algae.html
Oregon: Oregon Harmful Algae Bloom Surveillance Program, Oregon Health Authority (OR-HABS)	•Improved the quality of information provided to various audiences and to better respond to requests for guidance in developing policy and responding to events. •http://www.oregon.gov/DHS/ph/hab/index.html •Collaborated with the state Drinking Water Program to develop a HAB Communications Plan for public water systems, and other program partners and stakeholders to develop permanent HAB advisory signage that can provide general HAB messages to the public as well as cautionary messages related to specific HABs.
Virginia Department of Health (VDOH)	•Supported a website with outreach and education materials: •http://www.vdh.virginia.gov/epidemiology/DEE/HABS/ •Created the VDOH Algal Bloom Monitoring Network that is active during the summer and fall. The network covers 44 recreational beaches and 60 shellfish harvesting sites; and responds to fish kills and request for water sample analysis.
Washington State Department of Health (WSDOH)	•Collaborated with Washington State Department of Ecology and local health jurisdictions to provide resources and technical assistance for Washington’s passive Harmful Algae Bloom (HAB) surveillance system. •http://www.doh.wa.gov/CommunityandEnvironment/Contaminants/BlueGreenAlgae •http://www.ecy.wa.gov/programs/wq/plants/algae/monitoring/index.html •Strengthened its work on the Regional Examination of Harmful Algae Blooms (REHAB) which involves the monitoring of 30 lakes in three Washington counties through the active bloom months of June-October. Developed public access to cyanotoxin data: •https://www.nwtoxicalgae.org/ •Developed recreational guidance values for microcystins, anatoxin-a, cylindrospermopsins and saxitoxins which are incorporated into a protocol for local health jurisdictions, lake managers, and other agencies to follow in the case of toxic blooms.
Wisconsin Department of Health Services (WI DOHS)	•Collected and disseminated harmful algae-related illness data and environmental data. Specifically, resources supported the redesign of an interactive website that citizens and local health departments can use to report human or animal illnesses related to algal blooms in Wisconsin. This has resulted in the investigation of 102 human illness reports, and nine animal illness reports to date. •http://www.dhs.wisconsin.gov/eh/bluegreenalgae •Conducted active surveillance using an interactive website through a partnership with Poison Control Centers.

## 4. Discussion

Addressing the potential public health issues associated with the presence of toxin-producing cyanobacteria and algae in our drinking and recreational waters is an ongoing challenge because the risks associated with exposure vary across organisms, toxins, and routes of exposure. HABISS represented the first broad-scale attempt to conduct surveillance for human and animal health events and collect associated environmental data describing cyanobacteria and algae blooms in the U.S.

One of the key accomplishments of the HABISS program was the creation of partnerships among state-level health and environment departments. To complete HABISS data entry, NCEH’s public health partners needed access to environmental data typically collected by another entity, such as the department of environmental protection or environmental quality. Existing relationships across various departments were strengthened in some states, while others had to build new relationships to access the needed environmental data.

HABISS-related data collection allowed states to begin to understand the public health burden from human and animal exposures to cyanobacteria and algae blooms. For 2007–2011, 11 states reported over 4500 bloom events, 458 cases of suspected and confirmed human bloom-associated illnesses, and 175 animal morbidity and mortality events. Over half (248 of 458) of the human illnesses were ciguatera fish poisonings (CFP). CFP cases from outbreaks should be captured by CDC’s ongoing Foodborne Disease Outbreak Surveillance System (FDOSS) [4]. Individual CFP cases would not be captured in FDOSS; however, they could be captured in HABISS.

We anticipated many reports of adverse health outcomes (e.g., respiratory irritation, asthma exacerbations) associated with Florida red tides based on the work of the Red Tide Research Group [12,13,14]. However, thousands of beach-goers may be adversely affected by aerosolized toxins generated during Florida red tides, and it is not possible to accurately record the number of people with health-related complaints. Further, we understand the mechanism of the biological response to brevetoxins [15] and Florida has developed outreach and education materials to help beach-goers understand the risks associated with visiting the beach or bathing while there is an ongoing Florida red tide (see the website of the Florida Fish and Wildlife Conservation Commission [16]). Rather than marine waters, fresh waters were the source of exposure in nearly all (176 of 183, 96%) the adverse human health outcomes reported to HABISS.

There are few clinical data describing adverse health effects from exposure to cyanobacteria and associated toxins, making the clinical differential diagnosis difficult. The human and animal modules of HABISS were designed to collect specific information about signs and symptoms from exposure to cyanobacteria, including self-reported data. About half of the human cases reported dermatologic symptoms following exposure to cyanobacteria blooms, while much smaller numbers reported gastrointestinal or neurologic symptoms. This makes sense as over 90% of the exposures occurred during recreational activities involving direct contact with the water, such as swimming, *i.e*., dermal contact could produce rashes, and swallowing water during swimming could induce gastrointestinal and/or neurologic effects. However, in reviewing the HABISS data, no clear clinical picture of cyanobacteria- and cyanotoxin-related illnesses emerged. Additional epidemiologic and detailed clinical information describing the health effects associated with exposure to freshwater cyanobacteria and algae is needed to create more specific case definitions.

Although we cannot specifically identify which cyanobacteria produced the toxins reported in HABISS, the cyanobacteria cell counts and concentrations provide some interesting findings (Table 4). For *Anabaena* spp., the cell counts in waters associated with a health event, a fish kill, or a bloom event were all of the same order of magnitude (tens of thousands of cells/mL). However, the median concentration of anatoxin-a (produced by *Anabaena flos-aquae* and *A. lemmermannii* [17]) was an order of magnitude greater in samples collected in response to a health event when compared with the concentrations in water samples collected in response to a sighted bloom. Our results are consistent with the fact that not all cyanobacteria blooms produce toxin and the idea that an A*nabaena* bloom may be visible before it produces enough toxin to be a health threat.

There were few samples collected in response to an event that were analyzed for *Cylindrospermopsis* spp., perhaps because this species does not form floating scums, but tends to concentrate well below the water surface [18]. Six samples were collected in response to a health event and 5 were collected in response to a bloom report. These samples were analyzed for cylindrospermopsins, which are produced by *Anabaena*, *Aphanizomenon*, and *Cylindrospermopsis* spp. A higher median concentration of cylindrospermopsins was found in samples collected in response to a health threat than in samples collected in response to a bloom report.

Cell counts from 63 samples were reported for *Microcystis*, *Plantothrix*, and *Oscillatoria* spp., all of which can produce microcystins [18]. The median concentrations for total microcystins and microcystin L-R were higher in samples collected in response to a health event than for those collected in response to a bloom report, but the maximum concentration (120,000 µg/L) was much higher for the bloom response samples. While there are some trends in the cell counts and toxin concentrations, more data are needed to begin to extrapolate from these values to potential adverse health outcomes from specific blooms.

An electronic system to capture new cyanobacteria and algae bloom-related human and animal health and environmental data will be available through CDC’s NORS. Although NORS is a passive surveillance activity, CDC requires all states to report all disease “outbreaks” (defined as more than one case of a disease epidemiologically linked in space and time). Several states have attempted to enter HAB-related outbreak data using existing NORS forms but encountered difficulties in entering toxin concentrations and relevant symptoms because the system is currently designed to report infectious disease outbreaks. We expect improved HAB-related illness outbreak reporting for both people and animals from all states once the module specifically designed to capture these data becomes available.

In addition to access to a standardized surveillance system, the HABISS program provided resources to state partners to mitigate the public health impacts from cyanobacteria and algae blooms. States had a number of challenges in creating outreach and education materials about the potential risks from cyanobacteria and algae for the public. Many popular lakes experience blooms every summer; and it was difficult to craft prevention messages warning about risks from an event that has occurred and been observed many times without apparent associated illness outbreaks. Signs warning the public about blooms caused confusion when they are placed to identify a toxin-producing bloom occurring near, but not affecting, a swimming beach. Warnings are sometimes ignored by swimmers and others using waterbodies for recreational activities. Citizen groups that support monitoring activities caused confusion if they reported the presence of toxins found only in visible scums that are typically avoided by people. By contrast, people who live near lakes that bloom often may have a history of experiencing health symptoms, such as respiratory or eye irritation, and stay away from the water during blooms.

The data in HABISS reflects recreational exposures for humans and domestic pets. However, large surface waters that provide drinking water to towns and cities are also at risk for cyanobacteria blooms that could result in disease outbreaks. For example, the 2014 *Microcystis aeruginosa* bloom in Lake Erie resulted in measurable, but low, levels of microcystins in Toledo, Ohio’s drinking water. Nearly 500,000 people were affected by the Ohio EPA’s “Do not boil, do not drink” order. Toledo was able to quickly restore water to its distribution system, however, smaller community drinking water systems may lack resources needed to prevent or respond quickly to a large bloom in their water source [19].

People continue to ask local health and environmental experts the following questions about the risks associated with using a waterbody with an ongoing bloom: Can I swim in the water during a bloom?, Is my drinking water safe?, Can I eat the fish I catch?, Can I let my child or dog play in the water?, Why does my lake stink?, and “I bought this house so I could enjoy lakeside living--how do I clean up my lake? Answers to these questions comprise public and environmental health considerations, and many states have developed guidelines for informing the public about risks from cyanobacteria blooms, including risks from direct water contact, swallowing untreated water, and eating fish [20]. These efforts demonstrate that exposure to cyanobacteria blooms and associated toxins is a national public health issue needing ongoing attention.

Despite increasing awareness of HABs we can assume that there is under-reporting of possible cyanobacteria- and algae-related illnesses. Under-reporting is an issue with public health surveillance in general because ill people may not report the event to a health care practitioner, or the practitioner may not make the correct diagnosis. Even with a correct diagnosis, diseases are not typically reported to local or state public health departments for surveillance unless specific legislation requires reporting [1]. Reporting of illnesses suspected or confirmed to be associated with an environmental exposure is even less likely to occur because many state health departments lack resources to identify and follow up these events to collect and analyze appropriate environmental samples.

In addition to the problem of under-reporting, there are a number of other limitations with HABISS data. The system was available for data entry for a short time. The number of reports tended to increase over time, partly because public health practitioners established links with environmental health practitioners, allowing them to exchange environmental and health information about these events and partly because outreach activities encouraged reporting. HABISS collected data on acute events, and we were not able to capture cases with chronic effects. One way to address this would be to create a data-sharing partnership with another state or national system, such as the National Poison Data System (NPDS), which receives calls from people with chronic complaints as well as those with acute illnesses. As part of outreach efforts, State Health Departments can encourage physicians to report cases involving chronic health outcomes, such as neurologic effects sometimes associated with ciguatera fish poisoning or liver toxicity associated with exposure to microcystins, to the HAB module of NORS. Finally, clinical data are needed to inform and improve case definitions. Many of these limitations will be addressed by new data collected as part of the updated NORS. For example, NORS is a familiar surveillance system and contributing data to a new module of an existing system would be less burdensome than contributing to an independent surveillance system such as HABISS. Because of our experience with HABISS, the HAB module in NORS comprises variables that reflect data, such as exposure location, exposure routes, and signs and symptoms of exposure, available to state health departments.

## 5. Summary and Conclusions

Although potential health hazards associated with the toxins produced by cyanobacteria and algae have been known for decades or more, identifying individual cases of many of the illnesses produced by exposure to these toxins remains a diagnosis of exclusion accompanied by detailed information about the patient’s recent exposure history. States entered 458 suspected or confirmed human illnesses and 175 animal morbidity and mortality cases into HABISS associated with bloom events during 2007–2011. The information recorded in HABISS and the application of these data to develop a wide range of public health prevention and response activities indicate that cyanobacteria and algae blooms are an environmental public health issue that needs continuing attention at local, state, and national levels. Going forward, we will use the NORS module to track the frequency and geographic extent of cyanobacteria and algae blooms that affect public health.
